# Timing matters: tacrolimus intra-patient variability within the initial seven months forecasts *de novo* DSA and subsequent rejection in a Chinese kidney transplant cohort

**DOI:** 10.3389/fimmu.2026.1809202

**Published:** 2026-05-11

**Authors:** Xuebin Wang, Jia You, Hong Sun, Dan Wu, Hongxia Liu, Yan Zhu, Lizhi Chen, Jiazhao Fu, Wenyu Zhao, Mingxing Sui

**Affiliations:** 1 Department of Pharmacy, Shanghai Children’s Hospital, School of Medicine, Shanghai Jiao Tong University, Shanghai, China; 2 School of Pharmacy, Bengbu Medical University, Bengbu, Anhui, China; 3 Bioinformatics Center, Shanghai Children’s Hospital, School of Medicine, Shanghai Jiao Tong University, Shanghai, China; 4 Shanghai Altimetria Information Technology LLC, Shanghai, China; 5 Department of Organ Transplantation, Shanghai Changhai Hospital, Naval Medical University, Shanghai, China

**Keywords:** antibody-mediated rejection, *de novo* donor-specific antibody, intra-patient variability, kidney transplantation, tacrolimus

## Abstract

**Background:**

High intra-patient variability (IPV) in tacrolimus exposure is a known risk factor for poor long-term kidney transplant outcomes. However, significant heterogeneity exists regarding the optimal post-transplant period for its assessment. This study aimed to identify the earliest predictive time window for tacrolimus IPV and to evaluate its association with *de novo* donor-specific antibody (dnDSA) development and rejection, while also examining the impact of CYP3A5*3 and CYP3A4*1G polymorphisms on IPV.

**Methods:**

In a single-center retrospective study (2016-2021) of Chinese kidney transplant recipients, tacrolimus IPV (coefficient of variation) was calculated across consecutive intervals from transplantation to month 24, using all available whole blood drug trough concentration measurements within each window. The primary analysis assessed the association between time-window-specific IPV and the cumulative 7-year incidence of dnDSA, antibody-mediated rejection (ABMR), and T-cell-mediated rejection (TCMR). The influence of dnDSA on rejection and of genetic polymorphisms on IPV was also analyzed.

**Results:**

A total of 446 kidney transplant recipients were included in the analysis. The cumulative incidences of dnDSA and overall rejection were 16.37% (73/446) and 17.04% (76/446), respectively, comprising ABMR (n=49, 64.47%), TCMR (n=21, 27.63%), and mixed ABMR + TCMR (n=6, 7.89%). Tacrolimus IPV calculated over the first 7 postoperative months emerged as the earliest significant predictor for dnDSA development (*P* = 0.0048). IPV from month 13 onward significantly correlated with overall rejection risk (*P* = 0.014). No association was found between CYP3A5*3 or CYP3A4*1G polymorphisms and IPV. Furthermore, dnDSA-positive patients had significantly higher risks of overall rejection (34.25% vs. 10.99%, *P* = 0.022) and ABMR (26.03% vs. 8.04%, *P* = 0.0049) compared to dnDSA-negative patients.

**Conclusion:**

Tacrolimus IPV during the initial 7 post-transplant months serves as the earliest predictive window for dnDSA, a key risk factor for subsequent ABMR. These findings advocate for a dual strategy integrating early IPV-guided tacrolimus monitoring and systematic dnDSA surveillance to improve long-term graft outcomes.

## 1 Introduction

Kidney transplantation remains the optimal treatment for end-stage renal disease (1), yet long-term allograft survival is a persistent challenge, highlighting the critical need for personalized immunosuppressive management (2). As the cornerstone of maintenance immunosuppression, used in over 90% of kidney transplant recipients (3, 4), tacrolimus presents a narrow therapeutic index and significant intra-patient variability (IPV). This variability frequently results in periods of subtherapeutic or supratherapeutic exposure during long-term follow-up, increasing the risk of adverse outcomes including graft failure (5).

Tacrolimus IPV, defined as fluctuations in whole-blood trough concentrations within an individual (6), arises from a complex interplay of factors. These include genotype, ethnicity, drug formulation, patient non-adherence, dosage adjustments, drug-drug/food interactions, and gastrointestinal events (7, 8). Genetic polymorphisms in metabolizing enzymes, particularly CYP3A5 and CYP3A4, contribute substantially. In the Chinese Han population, the mutational frequencies of CYP3A5*3 (rs776746) and CYP3A4*1G (rs2242480) are approximately 70% and 20%, respectively (9, 10). However, evidence regarding the impact of the CYP3A5*3 polymorphism on tacrolimus IPV is inconsistent, and the influence of CYP3A4*1G remains understudied in China (11, 12). Notably, our recent work in a Chinese heart transplant cohort identified the CYP3A5 rs15524 GG and CYP3A7 rs776744 TT genotypes as significant predictors of higher early post-transplant tacrolimus IPV (13).

Critically, elevated tacrolimus IPV is a recognized risk factor for poor transplant outcomes across solid organ types, including kidney and liver transplants in both adults and children (14–16). Our prior research corroborates this, demonstrating that high tacrolimus IPV impairs long-term allograft function in kidney transplant recipients (17). Consequently, IPV has emerged as a valuable metric for therapeutic drug monitoring (18), as maintaining stable exposure is essential for minimizing rejection risk (19). Mounting evidence indicates that high IPV not only predisposes to early T-cell-mediated rejection (TCMR) but is also associated with the long-term development of *de novo* donor-specific antibodies (dnDSA) and antibody-mediated rejection (ABMR), ultimately jeopardizing graft survival (20–22).

A pivotal unanswered question centers on the timing of IPV assessment. While many studies in kidney transplantation calculate IPV using tacrolimus concentration data from 6 to 12 months post-transplant to predict the occurrence of dnDSA and long-term graft (5, 13, 23, 24), others suggest clinically relevant variability extends beyond 12 months (25). This heterogeneity underscores a lack of consensus on the optimal post-transplant period for IPV evaluation (5, 26). Identifying the critical window during which IPV most accurately predicts clinical risk is therefore essential. It remains unclear, particularly in Chinese populations, whether early post-transplant IPV holds prognostic value comparable to later measurements. Defining this timeline could refine monitoring strategies and enable timely intervention. Furthermore, establishing early IPV-based predictive models would offer significant clinical utility if early variability proves informative.

Currently, there is a paucity of studies comprehensively evaluating the correlation between tacrolimus IPV, dnDSA, and rejection episodes (encompassing both ABMR and TCMR) in Chinese kidney transplant recipients. This gap is particularly evident in studies that systematically investigate the predictive power of IPV across different post-transplant intervals, while also considering the potential moderating role of pharmacogenetic variants prevalent in this population, such as CYP3A5*3 and CYP3A4*1G. Therefore, this study aimed to investigate these relationships within a Chinese kidney transplant cohort. We sought not only to identify the earliest cumulative time window at which tacrolimus IPV exerts a significant warning effect on clinical outcomes but also to explore the influence of key genetic polymorphisms on IPV, thereby providing a more holistic view of the determinants of tacrolimus exposure variability and its clinical consequences.

## 2 Methods

### 2.1 Study design

This study is a retrospective, single center, non-interventional study.

### 2.2 Study population

The study population consisted of patients who underwent kidney transplantation at the Organ Transplantation Department of Shanghai Changhai Hospital between January 2016 and December 2021.

### 2.3 Inclusion and exclusion criteria

The inclusion criteria include: (1) Receiving a dual or triple immunosuppressive regimen of tacrolimus, MMF (or EC-MPS), and/or glucocorticoids after kidney transplantation; (2) Be able to obtain a dosing regimen for tacrolimus within 3–24 months after kidney transplantation, with at least 3 measurements of whole-blood tacrolimus trough concentration (C_0_); (3) Having complete clinical data and relevant follow-up data.

Exclusion criteria include: (1) Second kidney transplant; (2) Kidney transplantation combined with other organ transplantation; (3) Delayed recovery of transplanted kidney function; (4) Failure or death within 3 months after kidney transplantation; (5) Unable to obtain the IPV value of tacrolimus; (6) No results of dnDSA and preformed donor specific antibody (pfDSA) testing; (7) Unable to obtain serum creatinine test results for kidney transplant recipients.

### 2.4 Ethical approval

The research procedures comply with the 1964 Helsinki Declaration and its amendments. The research protocol has been approved by the Ethics Committee of Shanghai Changhai Hospital (CHEC2021-133), and all kidney transplant recipients participating in the study are exempt from informed consent.

### 2.5 Study population screening

878 kidney transplant recipients were retrospectively enrolled, who underwent kidney transplantation between January 2016 and December 2021.

**Figure 1:**
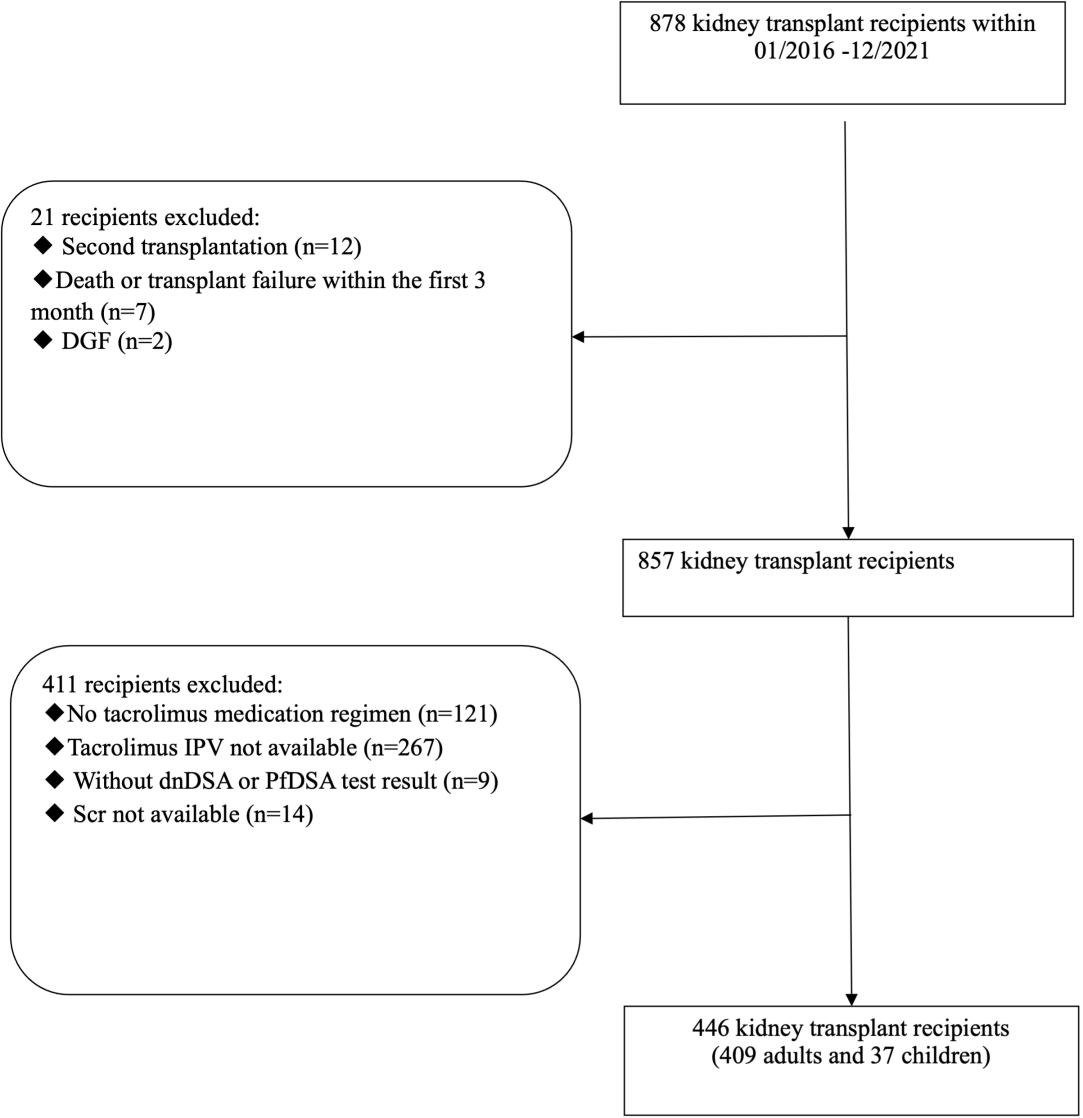
Flowchart of study population.

Based on the set inclusion and exclusion criteria, a total of 446 eligible individuals were screened. The detailed screening process was shown in **Figure 1**.

### 2.6 Clinical data collection

Based on integrated data from the Hospital Information System (HIS), Laboratory Information System (LIS), and the Therapeutic Drug Monitoring (TDM) information management system of the Clinical Pharmacy Department, a comprehensive set of medical information was collected. This included clinical medical records, transplant graft biopsy reports, donor-specific antibody (DSA) testing results, laboratory data, and medication history.

Immunosuppressive induction therapy comprised interleukin-2 receptor antagonists (IL-2RA, i.e., basiliximab), anti-thymocyte globulin (ATG), and the CD20 monoclonal antibody (rituximab). For postoperative maintenance immunosuppression, a combination regimen typically included tacrolimus along with mycophenolate mofetil (MMF) or enteric-coated mycophenolate sodium (EC-MPS), with or without glucocorticoids (prednisone acetate or methylprednisolone tablets). In some recipients, sirolimus (SRL) or mizoribine (MZR) was used in combination with or as a substitute for mycophenolic acid (MPA).

In this retrospective study, the diagnosis of rejection was primarily based on biopsy-confirmed findings and DSA testing. While this approach likely identified most cases of ABMR due to its reliance on histopathological and serological evidence, it may have led to underreporting of TCMR. Clinically mild TCMR episodes—often treated empirically with intensified immunosuppression in outpatient settings without biopsy verification—may have been overlooked. This could explain the relatively low incidence of TCMR (4.71%) observed in our cohort compared to expected rates (>30% reported in prospective studies). This methodological limitation should be taken into account when interpreting findings related to TCMR.

### 2.7 Calculation of tacrolimus IPV

Tacrolimus IPV was calculated as the coefficient of variation (CV%) of multiple trough concentration (C_0_) measurements taken within consecutive time windows, with results expressed as the median and interquartile range. For each recipient, all available C_0_ measurements from transplantation through the end of a given postoperative month were included in the corresponding window, provided at least three measurements were available.

For each window, IPV was calculated as: $CV\%\;=\frac{SD(C_{0})}{Mean(C_{0})}\times \;100\%$ (27).

Each window was defined as “Months 0 to X,” where X ranged from 4 to 24 months. For instance, the “4−month IPV” included all available C_0_ measurements from the day of transplantation through the end of the fourth postoperative month. Using this approach, twenty−one distinct windows were evaluated, spanning from the immediate post−transplant period up to Month 24. This cumulative-window design was used to identify the earliest post-transplant period at which tacrolimus IPV became clinically informative for subsequent dnDSA and rejection outcomes.

### 2.8 Determination of tacrolimus blood concentration

Peripheral venous blood (0.5–1 mL) was collected from each kidney transplant recipient approximately 30 minutes before drug administration and placed into a 1−mL EDTA−K_2_ anticoagulant tube. Samples were delivered to the TDM laboratory of the pharmacy department before 09:00 daily. Tacrolimus trough concentrations (C_0_) were measured using a chemiluminescence microparticle immunoassay (CMIA) with the Architect Tacrolimus quantitative detection kit (Abbott Diagnostics, Lake Forest, IL, USA), according to the manufacturer’s instructions.

In addition, with reference to the *Expert Consensus on Individualized Treatment of Tacrolimus in Solid Organ Transplantation* and based on clinical transplantation practice at our center, the monitoring frequency for tacrolimus trough concentrations was defined as follows: 2–4 times per week within the first postoperative month, 1–2 times per week during the second month, 1–2 times per month between months 3 and 6, and once every quarter or annually thereafter.

### 2.9 Genotyping

Collected whole−blood samples were placed in EDTA−K_2_ anticoagulant tubes and refrigerated at 4°C for up to 8 hours. Samples were then transferred to −20°C for short−term storage; those intended for preservation beyond one month were subsequently moved to −80°C for long−term cryopreservation. SNP genotyping was performed using the SNaPshot™ multiplex system (Applied Biosystems). PCR primers were designed with Primer 5 software. Purified PCR products were subjected to SNaPshot primer extension. Alleles were resolved on an ABI 3730xl DNA analyzer and analyzed with GeneMapper^®^ 4.0 software.

### 2.10 Statistical analysis

Statistical analyses were conducted using SPSS software (version 23, IBM Corp., Armonk, NY, USA). Normality of data was assessed by the Kolmogorov–Smirnov or Shapiro–Wilk test, as appropriate. Continuous variables following a normal distribution are expressed as mean ± standard deviation ( $\bar{x}$ ± SD) and compared between groups using the independent-samples t−test. Non−normally distributed continuous variables are presented as median with interquartile range (IQR) and analyzed with the Mann–Whitney U or Kruskal–Wallis H−test, as indicated. Categorical variables are summarized as frequency and percentage. Survival analysis was performed using the Kaplan–Meier method, with between−group comparisons evaluated by the log−rank test. This approach was applied to assess the association of tacrolimus IPV with the cumulative incidence of dnDSA, ABMR, and TCMR, as well as to examine the impact of dnDSA on subsequent ABMR and TCMR occurrence. To determine the critical predictive window of IPV, we systematically evaluated the relationship between tacrolimus IPV—calculated across sequential time windows from Month 0 to Month 24—and the 7−year post−transplant cumulative incidence of dnDSA, ABMR, and TCMR. A two−sided P−value <0.05 was considered statistically significant.

## 3 Results

### 3.1 Clinical basic information

According to the inclusion and exclusion criteria, a total of 446 kidney transplant recipients were enrolled, comprising 409 adults and 37 children. Within 7 years post-transplantation, the cumulative incidence of dnDSA was 16.37% (73 cases). The cumulative incidence of allograft rejection (including ABMR and TCMR) was 17.04% (76 cases), with incidences of ABMR and TCMR being 10.99% (49 cases) and 4.71% (21 cases), respectively. Notably, 6 recipients experienced both ABMR and TCMR. The detail information was shown in **Table 1**.

**Table 1:** Baseline clinical characteristics of kidney transplant recipients (n=446).

Category	Description	Total values	Values	
			Adults (n=409)	Children (n=37)
Baseline characteristics	Age (years)	40 (30, 49)	41 (32, 50)	12 (11, 15.5)
	Male	265 (59.4%)	244 (59.7%)	21 (56.8%)
	Weight (kg)	60 (30, 49%)	68 (60.4, 75.70)	35 (24.9, 45.35)
	BMI (kg/m^2^)	21.48 ± 3.69	21.46 (19.6, 24.02)	15.8 (14.47, 18.8)
Source of donor	Deceased donor	392 (87.89%)	355 (79.59%)	37 (8.3%)
	Living donor	54 (12.11%)	54 (12.11%)	0
Donor specific antibody	pfDSA	35 (7.85%)	33 (7.40%)	2 (0.45%)
	Class I pfDSA	24 (5.38%)	23 (5.17%)	1 (0.21%)
	Class II pfDSA	22 (4.93%)	20 (4.48%)	2 (0.45%)
	Class I and II pfDSA	11 (2.47%)	10 (2.24%)	1 (0.03%)
	dnDSA	73 (16.37%)	64 (14.35%)	9 (2.02%)
Type of dialysis	Hemodialysis	241 (54.04%)	225 (50.45%)	16 (3.59%)
	Peritoneal dialysis	155 (34.75%)	138 (30.94%)	17 (3.81%)
	Hemodialysis and peritoneal dialysis	42 (9.42%)	40 (8.97%)	2 (0.45%)
	Unknown	8 (1.79%)	6 (1.35%)	2 (0.45%)
Rejection, n (%)	Total rejection	76 (17.04%)	58 (13%)	8 (1.79%)
	TCMR	21 (4.71%)	19 (4.26%)	2 (0.45%)
	ABMR	49 (10.99%)	45 (10.09%)	4 (0.9%)
	TCMR and ABMR	6 (1.3%)	6 (1.3%)	0
Inducing immunosuppressant	Anti-thymocyte globulin	361 (80.94%)	337 (75.56%)	24 (5.38%)
	Basiliximab	93 (20.85%)	83 (18.61%)	10 (2.24%)
	Rituximab	1 (0.22%)	1 (0.22%)	0
Tacrolimus IPV	CV%	21.7 (17.4, 26)	21.5 (17.4, 25.8)	23.2 (19.2, 28.45)
Primary kidney disease	Hypertensive nephropathy	176 (39.46%)	167(37.44%)	9 (2.02%)
	Glomerulopathy	42 (9.42%)	37 (8.3%)	5 (1.12%)
	IgA nephropathy	41 (9.19%)	40 (8.97%)	1 (0.22%)
	Polycystic kidney	14 (3.14%)	11 (2.47%)	3 (0.67%)
	Diabetic nephropathy	13 (2.91%)	13 (2.91%)	0
	Focal segmental glomerulosclerosis	5 (1.12%)	5 (1.12%)	0
	Unknown	207 (46.41%)	185 (41.48%)	22 (4.93%)

Continuous data are presented as mean with SD or median with IQR, depending on variable distribution. ABMR, antibody-mediated rejection; BMI, body mass index; CV, coefficient of variation; dnDSA, *de novo* donor specific antibody; IPV, intra-patient variability; pfDSA, preformed donor specific antibody; TCMR, T cell mediated rejection.

### 3.2 Effect of gene polymorphisms on tacrolimus IPV

**Figure 2:**
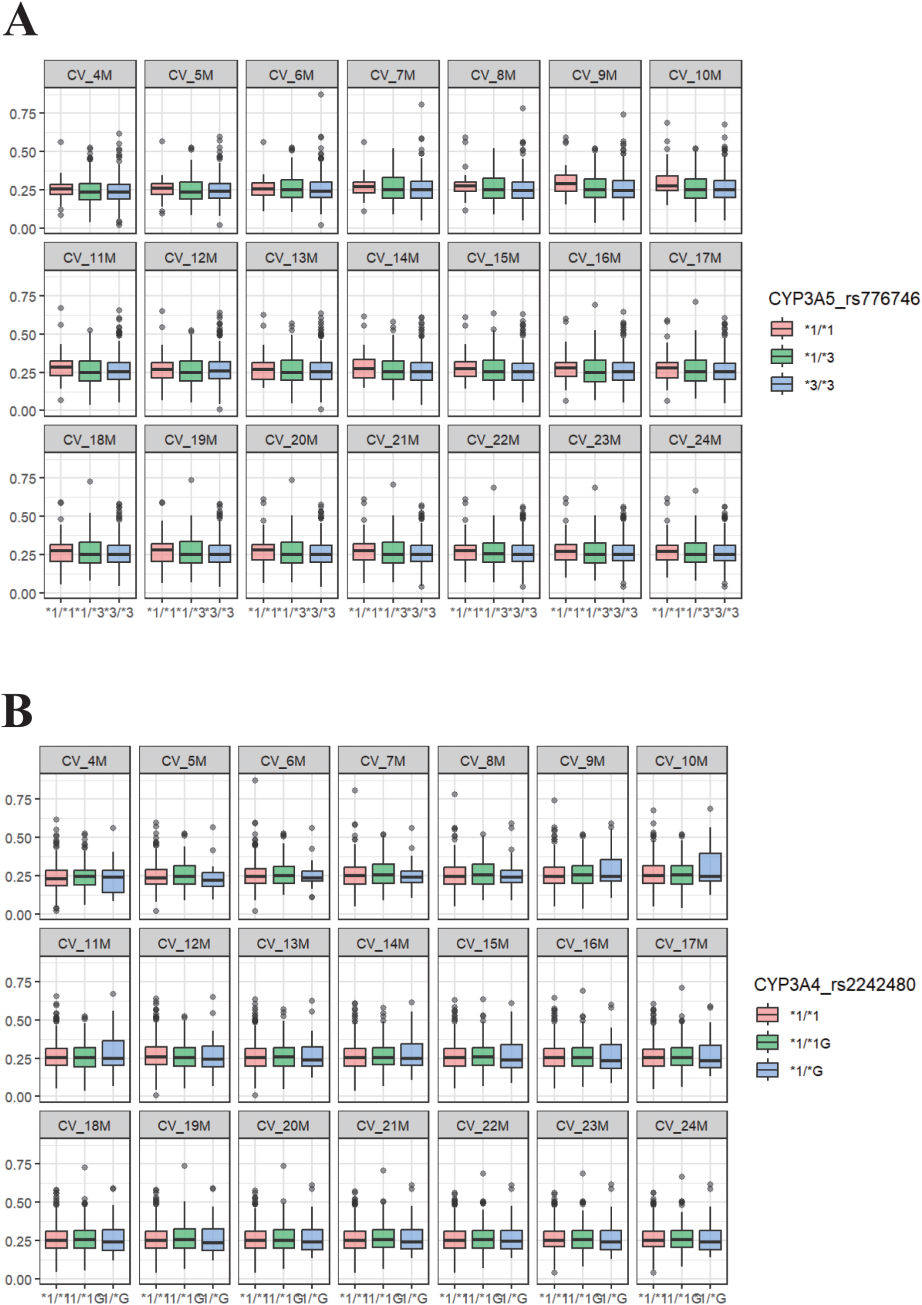
Gene polymorphism not correlated with tacrolimus IPV at different stages within 24 months after kidney transplantation (n=446). **(A)** CYP3A5_rs776746, **(B)** CYP3A4_rs2242480. CV, coefficient of variation; M, months; Across all time points, the minimum ANOVA p−values were 0.202 for CYP3A5_rs776746 and 0.188 for CYP3A4_rs2242480, indicating no statistically significant association at any post−transplant stage.).

Genetic testing was performed in 446 patients. To further investigate factors that may influence tacrolimus IPV, the impact of CYP3A5*3 and CYP3A4*1G polymorphisms was analyzed over the first 24 months following transplantation. As shown in **Figure 2**, no significant association was observed between these genetic variants and tacrolimus IPV during this period.

### 3.3 Effect of tacrolimus IPV on dnDSA and rejection

**Figure 3:**
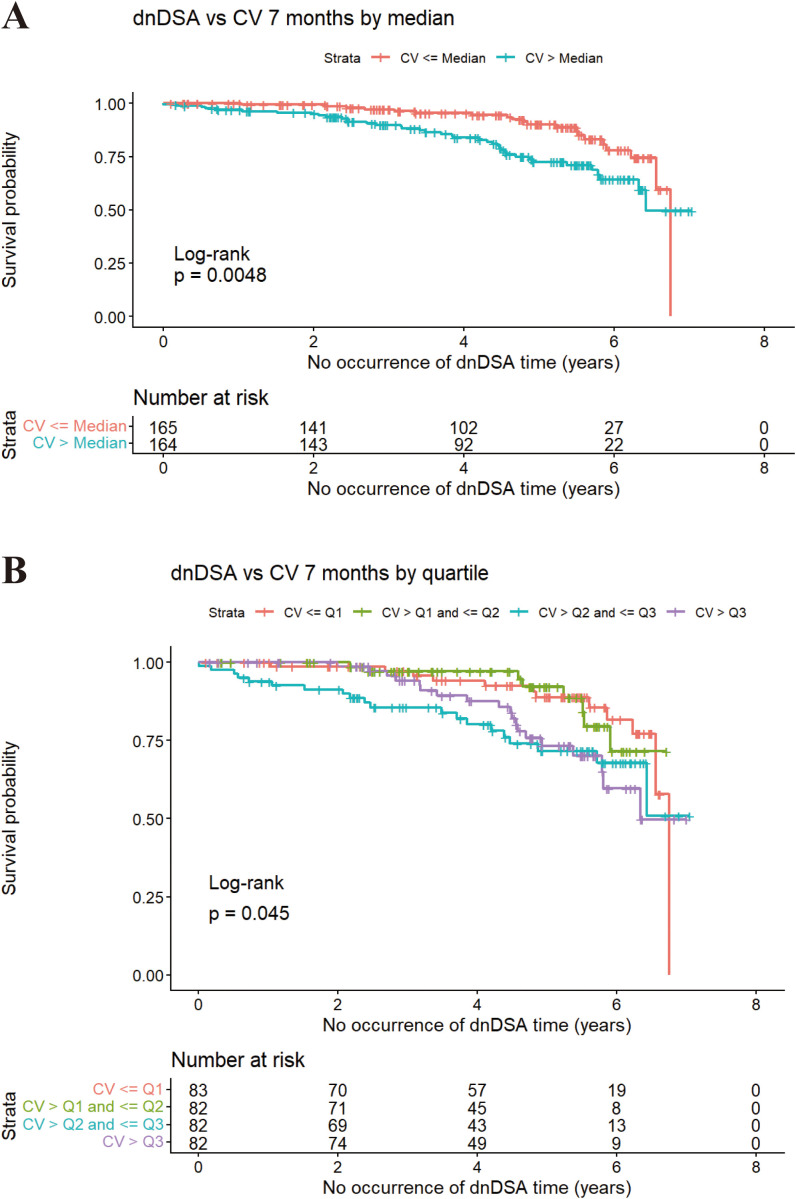
Effect of tacrolimus IPV on the occurrence of dnDSA in kidney transplant recipients at 7 months (n=446). **(A)** stratified by the median CV%, **(B)** stratified by the quartile CV%; dnDSA, *de novo* donor-specific antibodies; CV, coefficient of variation).

We evaluated the effect of tacrolimus IPV, stratified by median and quartiles of coefficient of variation (CV%) across post-transplant intervals within 2 years on 7-year clinical outcomes on 7−year clinical outcomes across different post−transplant intervals within the first 2 years (**Table 2**). During the initial 4–6 months post−transplantation, no significant association was observed between IPV and the development of dnDSA (*P* > 0.05). However, IPV calculated using all tacrolimus C_0_ levels from the first 7 months post−transplantation (0–7−month cumulative window) emerged as the earliest significant predictor. Stratification by the median CV% revealed a statistically significant difference in dnDSA−free survival between the two groups (*P* = 0.0048; **Figure 3A**). A comparable trend was observed with quartile−based stratification, although with slightly attenuated significance (*P* = 0.045; **Figure 3B**).

**Table 2:** The impact of tacrolimus IPV (by median and quartile) from the different months within 2 years after kidney transplantation on clinical outcomes of 7 years.

The cumulative PT for calculating IPV (months)	dnDSA *vs* CV (*P* value)		Rejection *vs* CV (*P* value)		ABMR *vs* CV (*P* value)		TCMR *vs* CV (*P* value)	
	by median	by quartile	by median	by quartile	by median	by quartile	by median	by quartile
4	**0.026**	0.11	0.72	0.8	0.85	0.86	0.082	0.21
5	**0.026**	0.17	0.98	0.69	0.91	0.81	0.41	0.44
6	0.061	0.25	0.61	0.68	0.88	0.68	0.33	0.25
7	**0.0048**	**0.045**	0.86	0.8	0.8	0.62	0.97	0.74
8	**0.00057**	**0.0071**	0.42	0.52	0.38	0.33	0.92	0.98
9	**0.003**	**0.028**	0.17	0.54	0.12	0.37	0.96	0.53
10	**0.00067**	**0.00055**	0.096	0.16	**0.021**	0.08	0.48	0.12
11	**0.00014**	**0.0045**	0.37	0.74	0.16	0.35	0.43	0.48
12	**0.00067**	**0.00017**	0.11	0.45	0.078	0.36	0.83	0.84
13	**<0.0001**	**<0.0001**	**0.014**	0.1	**0.027**	0.16	0.19	0.6
14	**<0.0001**	**<0.0001**	**0.0018**	**0.016**	**0.012**	0.067	0.15	0.42
15	**<0.0001**	**<0.0001**	**0.0046**	**0.04**	0.05	0.27	0.057	0.28
16	**<0.0001**	**<0.0001**	**0.0095**	0.077	0.1	0.43	0.12	0.39
17	**<0.0001**	**<0.0001**	**0.0049**	**0.026**	**0.0038**	0.18	0.071	0.12
18	**<0.0001**	**<0.0001**	**0.0062**	**0.0028**	**0.049**	0.12	0.15	0.34
19	**<0.0001**	**<0.0001**	**0.01**	**0.027**	0.08	0.12	0.15	0.16
20	**<0.0001**	**0.00035**	**0.014**	0.072	0.098	0.26	0.2	0.26
21	**<0.0001**	**0.00063**	**0.025**	0.072	0.083	0.23	0.41	0.47
22	**0.00028**	**0.0023**	**0.039**	0.086	0.21	0.27	0.22	0.3
23	**0.00018**	**0.0014**	**0.019**	0.061	0.21	0.071	0.19	0.25
24	**<0.0001**	**0.00086**	**0.041**	0.063	0.15	**0.047**	0.34	0.45

Pink, blue, and gray represent *P* < 0.01, *P* < 0.05, and *P*>0.05, respectively. ABMR, antibody-mediated rejection; CV, coefficient of variation; dnDSA, *de novo* donor specific antibody; IPV, intra-patient variability; PT, posttransplant time; TCMR, T cell mediated rejection.

Bold means P < 0.05.

Further sensitivity analysis was performed by excluding pediatric recipients, and the results showed no significant change in the overall findings (*P* = 0.0035). These results, which reinforce the robustness of the study, are shown in the **Supplementary Materials** (**Supplementary Figure 1**).

Regarding rejection outcomes, stratification by the median CV% showed progressively significant associations from month 7 onward. Statistically significant correlations were observed for overall rejection starting at month 13 (*P* = 0.014) and for ABMR at month 17 (*P* = 0.0038). And ABMR became significant as early as month 10 (*P* = 0.021). Notably, median−based stratification demonstrated stronger predictive performance for late−phase rejection events (beyond 12 months) compared with quartile categorization. In contrast, no consistent association was found between CV% and TCMR across most time intervals (*P* > 0.05).

### 3.4 Impact of dnDSA on rejection

**Figure 4:**
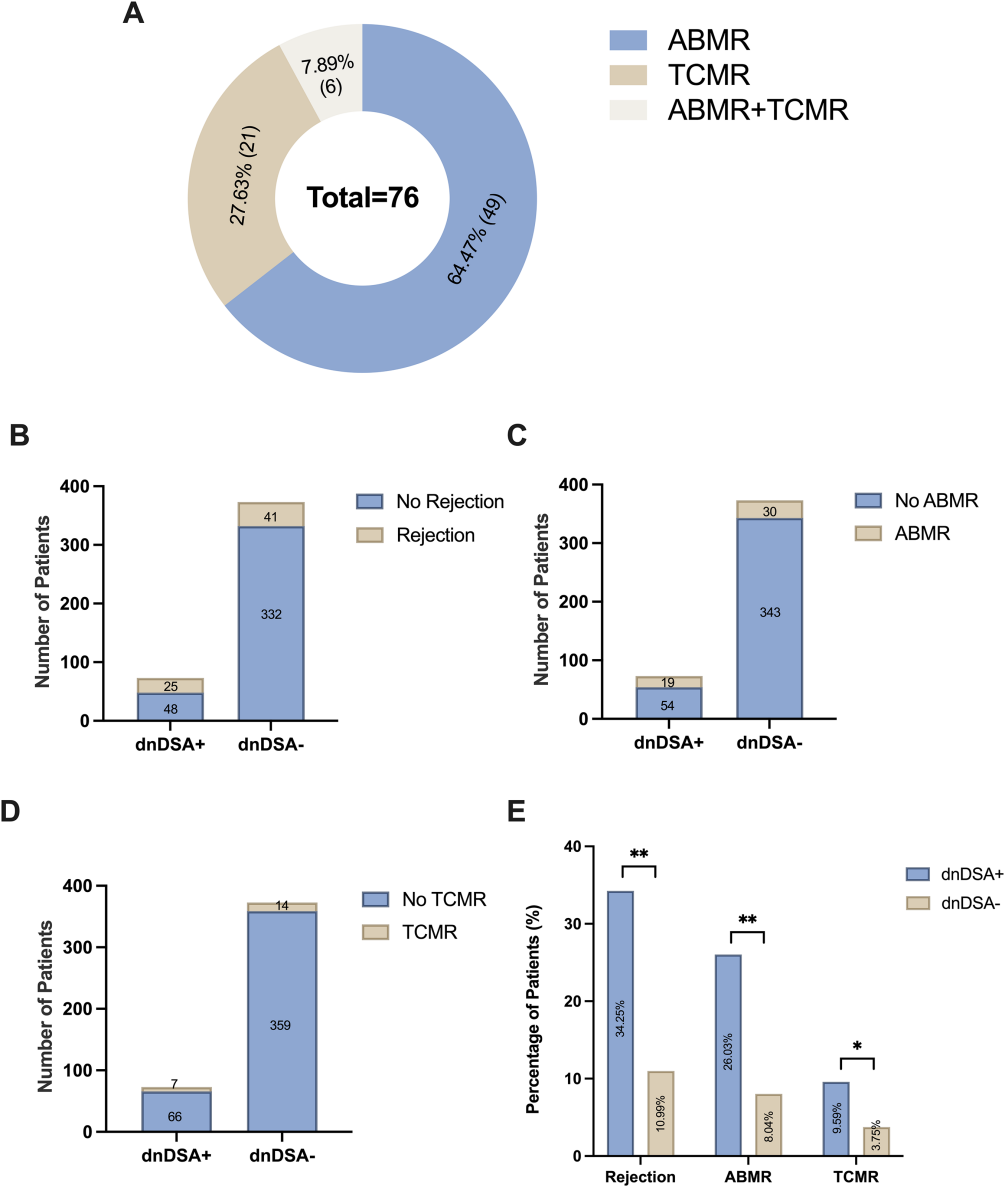
Effect of dnDSA on the occurrence of rejection in kidney transplant recipients (n=446). **(A)** Distribution of rejection types among patients; **(B)** Incidence of rejection in dnDSA positive and dnDSA negative patients; **(C)** Occurrence of ABMR in dnDSA positive and dnDSA negative patients; **(D)** Frequency of TCMR in dnDSA positive and dnDSA negative patients; **(E)** Comparative analysis of rejection rates between dnDSA positive and dnDSA negative patients; ABMR, ABMR: antibody-mediated rejection; TCMR, T cell mediated rejection; dnDSA, *de novo* donor-specific antibodies). The symbols represent statistical significance: * for P < 0.05, ** for P < 0.01.

Among 446 kidney transplant recipients, a total of 76 experienced rejection within 7 years after transplantation, including ABMR (n=49; 64.47%), TCMR (n=21; 27.63%), and mixed ABMR + TCMR (n=6; 7.89%), as shown in **Figure 4A**. Of these recipients, 16.37% (n=73) were dnDSA positive (+) and 83.63% (n=373) were dnDSA negative (-), respectively (**Figures 4B–D**).

**Figure 5:**
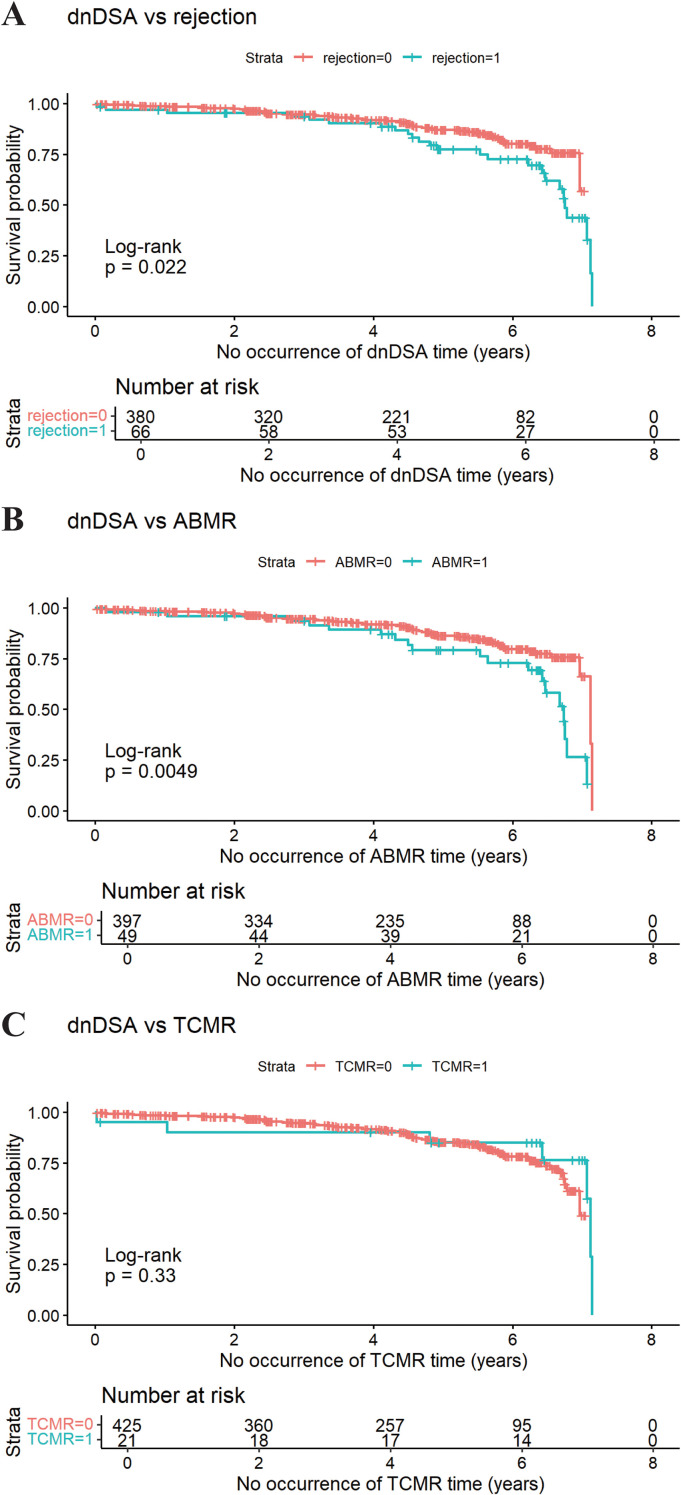
Kaplan–Meier analysis of rejection outcomes according to dnDSA status. **(A)** Overall rejection-free survival in dnDSA-positive versus dnDSA-negative recipients; **(B)** ABMR-free survival in dnDSA-positive versus dnDSA-negative recipients; **(C)** TCMR-free survival in dnDSA-positive versus dnDSA-negative recipients).

Firstly, we investigated the correlation between dnDSA and the cumulative incidence of overall rejection (including both ABMR and TCMR) in kidney transplant recipients at 7 years post-transplantation. The cumulative incidence of overall rejection was 34.25% (25 cases) in the dnDSA-positive group and 10.99% (41 cases) in the dnDSA-negative group, respectively (**Figures 4B, E**). Kaplan–Meier survival analysis demonstrated that the incidence of rejection was significantly higher in the dnDSA-positive group compared with the dnDSA-negative group (*P* = 0.022; **Figure 5A**).

Subsequently, subgroup analysis was performed for ABMR. The cumulative incidence of ABMR at 7 years post-transplantation was 26.03% (19 cases) in the dnDSA-positive group and 8.04% (30 cases) in the dnDSA-negative group, respectively (**Figure 4C**). Survival analysis indicated that the occurrence of ABMR was significantly higher in the dnDSA-positive group than in the negative group (*P* = 0.0049; **Figure 5B**).

Finally, subgroup analysis was conducted for TCMR. The cumulative incidence of TCMR at 7 years post-transplantation was 9.59% (7 patients) in the dnDSA-positive group and 3.75% (14 patients) in the dnDSA-negative group, respectively (**Figure 4D**). Kaplan–Meier analysis revealed no statistically significant difference in the cumulative incidence of TCMR between the dnDSA-positive and dnDSA-negative groups (9.59% vs. 3.75%, P = 0.33), although a numerical trend toward a higher incidence was observed in the dnDSA-positive cohort (**Figure 5C**).

## 4 Discussion

This retrospective single-center study investigated the association between whole-blood tacrolimus IPV, dnDSA development, and rejection in a cohort of 446 Chinese kidney transplant recipients. The main contribution of our study is not to re-demonstrate that high IPV and dnDSA are clinically relevant - this has been shown previously - but to clarify when cumulative tacrolimus IPV first becomes informative in an adult-dominant Chinese cohort. By systematically evaluating multiple post-transplant intervals, we identified that IPV calculated over the cumulative 0–7 month window represents the earliest time period during which IPV emerges as a robust and persistent risk factor for subsequent dnDSA development. Furthermore, dnDSA positivity was strongly associated with an elevated risk of ABMR, reinforcing its established role as a key driver of alloimmune injury. We therefore view the present study as a timing- and population-specific extension of prior evidence rather than a mechanistic breakthrough.

First, we found no significant association between CYP3A5*3 or CYP3A4*1G polymorphisms and tacrolimus IPV within the first 24 months post-transplantation, which is consistent with prior reports. Nuchjumroon et al. demonstrated that CYP3A5 polymorphisms did not influence tacrolimus IPV 6–12 months after kidney transplantation in a Thai population (28), and Choi et al. similarly reported no significant effect in pediatric kidney transplant recipients (12). While CYP3A5 genotype is a well-known determinant of mean tacrolimus exposure across different transplant types (29–31), IPV reflects variability around this mean, which is primarily influenced by modifiable factors such as medication adherence, drug interactions, and gastrointestinal absorption (14, 32). These dynamic factors likely overshadow the impact of static genetic factors on IPV. Interestingly, our recent study in Chinese heart transplant recipients identified CYP3A5 rs15524 and CYP3A7 rs776744 genotypes as significant predictors of higher early post-transplant tacrolimus IPV (13), suggesting that certain rare or organ−specific genetic variants may still influence IPV. Further research is warranted to explore the potential influence of other genetic variants on tacrolimus IPV and dnDSA development in kidney transplantation.

A pivotal finding of our study is the identification of the specific post-transplant period during which tacrolimus IPV begins to significantly predict dnDSA development. Currently, no consensus exists regarding the optimal window for calculating IPV, which limits its standardized clinical application (5). While many studies have focused on the 6–12 month period (18, 33), often under the assumption of a stable dosing regimen to minimize confounding from early dose adjustments, our study adopted a different approach. We did not distinguish variability arising from dose changes from that due to other factors such as adherence or absorption. We found that IPV calculated over the first 7 months post-transplant was the earliest interval to emerge as a robust and persistent predictor of dnDSA development occurring from month 7 to year 7. Although sporadic significance was observed with IPV as early as 4 months (*P* = 0.026), the association became consistently strong and significant starting at the 7-month IPV (*P* = 0.0048), with both median and quartile stratifications demonstrating a higher cumulative incidence of dnDSA in patients with high IPV.

We interpret the 7−month IPV as a clinically meaningful transition point from the perioperative phase to a more stable maintenance period. By approximately month 7, most recipients have entered a stable maintenance phase, where persistently elevated IPV more specifically captures long−term risk factors such as nonadherence, inconsistent absorption, or interacting medications (14, 34). Repeated fluctuations during this phase may facilitate intermittent underimmunosuppression, thereby promoting alloimmune priming and subsequent dnDSA development. Future mechanistic studies are warranted to validate these hypothesis−generating observations.

Importantly, the clinical relevance of IPV may extend beyond whole−blood concentration variability. Our recent prospective study indicated that monitoring IPV in peripheral blood mononuclear cells (PBMCs)—the target cells of tacrolimus—may offer superior clinical insight (35). We observed that dnDSA-positive patients exhibited significantly higher IPV in PBMC tacrolimus levels compared to dnDSA-negative patients, a pattern not evident in whole-blood analyses. Although our current study did not dissect the sources of variability, the 7-month IPV offers a clinically practical and earlier predictive window for identifying patients at heightened risk of alloimmune activation.

Beyond its association with dnDSA, tacrolimus IPV also demonstrated a significant time-dependent relationship with rejection outcomes, particularly ABMR. Our analysis revealed that elevated IPV became significantly associated with overall rejection beginning at month 13 post-transplant. More specifically, a significant correlation with ABMR emerged as early as month 10 (*P* = 0.021; by median CV stratification), with consistently significant associations observed at multiple subsequent timepoints (months 13, 14, 17, and 18). The relationship between IPV and ABMR appears complex and may be modulated by immunological risk profiles. While Sablik et al. reported no difference in IPV between chronic active ABMR patients and controls (36), Kim et al. demonstrated that high IPV was linked to late-onset ABMR primarily in high-immunological-risk recipients (37). These discrepant findings may reflect variations in ABMR definitions, IPV calculation methodologies, and cohort risk profiles, underscoring the need for standardized approaches in future studies.

Our study further confirms a strong association between dnDSA positivity and ABMR. The cumulative incidence of ABMR was significantly higher in the dnDSA-positive group compared to the dnDSA-negative group, consistent with previous reports (18, 38, 39) and reinforcing dnDSA as a principal predictor of ABMR. However, not all dnDSA−positive patients in our cohort developed overt rejection. This discrepancy indicates that dnDSA may be best interpreted as a marker of heightened alloimmune risk rather than a direct surrogate for active, biopsy−proven rejection. As noted by Loucks−DeVos et al., only persistent dnDSA—as opposed to an isolated occurrence—significantly increases the risk of detrimental outcomes (40). In clinical practice, a positive dnDSA result could therefore prompt repeat testing to confirm persistence, review of tacrolimus IPV and adherence, monitoring of graft function and proteinuria, and consideration of biopsy if dnDSA persists or clinical abnormalities arise.

Interestingly, while we observed a numerical trend toward higher TCMR incidence in dnDSA-positive patients (9.59% vs. 3.75%), this difference did not reach statistical significance (*P* = 0.33). This finding is consistent with Liu et al. (41), who reported no significant difference in TCMR incidence between dnDSA-positive and negative patients but noted more frequent mixed rejection episodes among dnDSA-positive recipients. Importantly, the relatively low incidence of TCMR (4.71%) in our study likely reflects under-detection inherent to its retrospective design, which captured mainly moderate-to-severe cases requiring biopsy or hospitalization. Thus, the observed trend may indicate a real but underpowered association, and the relationship between dnDSA and TCMR remains uncertain, warranting further prospective evaluation.

When compared to international data, the cumulative incidence of ABMR in our adult recipients at 7 years post-transplant (10.09%) appears somewhat lower. Betjes et al. (42) reported that 10 years after kidney transplantation, the cumulative incidence of ABMR was 17%, 15%, and 12% in young (18–39 years), middle-aged (40–55 years), and elderly (>55 years) recipients, respectively (*P* < 0.001). This discrepancy may reflect differences in age distribution, immunosuppressive protocols, or ethnic factors between cohorts. Notably, Betjes et al. (42) found that ABMR incidence was age-dependent, plateaued later post-transplantation (8–10 years), and contributed minimally to graft loss in older recipients. These comparative observations, however, should be interpreted in light of our study’s inherent limitations.

Several limitations should be acknowledged. First, the single-center retrospective design may introduce selection bias and limit the generalizability of our findings. Second, the low number of TCMR events constrained the statistical power to analyze its relationship with IPV or dnDSA. Third, the primary analysis combined adults and children, potentially masking age-specific differences in drug handling, immunity, and rejection (42). Moreover, all pediatric recipients received deceased-donor kidneys, while living-donor transplants occurred only in adults—a known confounder of immunological outcomes. Thus, the findings should be interpreted at the overall-cohort level, and age-stratified validation in larger multicenter studies is needed. Future prospective multicenter research with protocol biopsies, standardized monitoring, and age-specific stratification is warranted to clarify these risk interactions.

Despite these limitations, our findings carry important clinical implications. The consistent predictive power of the 7-month IPV supports its use as a valuable early risk stratification tool. Implementing routine IPV assessment from this timepoint could help identify high-risk patients who may benefit from intensified counseling, adherence support, or closer serological monitoring for dnDSA. Furthermore, the strong link between dnDSA and ABMR reinforces the necessity of integrating systematic dnDSA surveillance with therapeutic drug monitoring into long-term transplant care protocols.

## 5 Conclusion

In summary, this study underscores the clinical significance of tacrolimus IPV in Chinese kidney transplant recipients. Our findings demonstrate that tacrolimus IPV is a critical determinant influencing the development of dnDSA and ABMR. Specifically, IPV calculated using tacrolimus concentration data from the first 7 months post-transplantation serves as the earliest reliable predictor for dnDSA risk emerging after month 7. Moreover, the presence of dnDSA is strongly associated with a substantially increased risk of ABMR, highlighting the imperative for combined monitoring of tacrolimus exposure stability and dnDSA status in clinical practice to mitigate rejection risks. Notably, our study suggests that the impact of common genetic polymorphisms (CYP3A5*3, CYP3A4*1G) on tacrolimus IPV may be limited, directing attention toward modifiable behavioral and pharmacological factors as primary targets for intervention. These insights contribute to defining an earlier, optimal risk-prediction window and support a strategy of integrated pharmacological and immunologic monitoring to improve long-term outcomes after kidney transplantation.

## Data Availability

The raw data supporting the conclusions of this article will be made available by the authors, without undue reservation.

## References

[1] Collins MG Fahim MA Pascoe EM Hawley CM Johnson DW Varghese J . Balanced crystalloid solution versus saline in deceased donor kidney transplantation (BEST-Fluids): a pragmatic, double-blind, randomised, controlled trial. Lancet. (2023) 402:105–17. doi: 10.1016/s0140-6736(23)00642-6. PMID: 37343576

[2] Robertson H Kim HJ Li J Robertson N Robertson P Jimenez-Vera E . Decoding the hallmarks of allograft dysfunction with a comprehensive pan-organ transcriptomic atlas. Nat Med. (2024) 30:3748–57. doi: 10.1038/s41591-024-03030-6. PMID: 38890530 PMC11645273

[3] Sommerer C Suwelack B Dragun D Schenker P Hauser IA Witzke O . An open-label, randomized trial indicates that everolimus with tacrolimus or cyclosporine is comparable to standard immunosuppression in de novo kidney transplant patients. Kidney Int. (2019) 96:231–44. doi: 10.1016/j.kint.2019.01.041. PMID: 31027892

[4] Lloberas N Grinyo JM Colom H Vidal-Alabro A Fontova P Rigo-Bonnin R . A prospective controlled, randomized clinical trial of kidney transplant recipients developed personalized tacrolimus dosing using model-based Bayesian prediction. Kidney Int. (2023) 104:840–50. doi: 10.1016/j.kint.2023.06.021. PMID: 37391040

[5] Baghai Arassi M Gauche L Schmidt J Hocker B Rieger S Susal C . Association of intraindividual tacrolimus variability with de novo donor-specific HLA antibody development and allograft rejection in pediatric kidney transplant recipients with low immunological risk. Pediatr Nephrol. (2022) 37:2503–14. doi: 10.1007/s00467-022-05426-3. PMID: 35166920 PMC9395307

[6] Schumacher L Leino AD Park JM . Tacrolimus intrapatient variability in solid organ transplantation: a multiorgan perspective. Pharmacotherapy. (2021) 41:103–18. doi: 10.1002/phar.2480. PMID: 33131078

[7] Mengel M Mannon RB . Banff and ABMR: are we going in the right direction? Am J Transplant. (2021) 21:2321–2. doi: 10.1111/ajt.16546. PMID: 33621422

[8] Chen H Liu S Yu L Hou X Zhao R . Factors and interventions affecting tacrolimus intrapatient variability: a systematic review and meta-analysis. Transplant Rev (Orlando). (2024) 38:100878. doi: 10.1016/j.trre.2024.100878. PMID: 39260119

[9] Hu GX Dai DP Wang H Huang XX Zhou XY Cai J . Systematic screening for CYP3A4 genetic polymorphisms in a Han Chinese population. Pharmacogenomics. (2017) 18:369–79. doi: 10.2217/pgs-2016-0179. PMID: 28244811

[10] Kuehl P Zhang J Lin Y Lamba J Assem M Schuetz J . Sequence diversity in CYP3A promoters and characterization of the genetic basis of polymorphic CYP3A5 expression. Nat Genet. (2001) 27:383–91. doi: 10.1038/86882. PMID: 11279519

[11] Cheung CY Chan KM Wong YT Chak WL Bekers O van Hooff JP . Impact of CYP3A5 genetic polymorphism on intrapatient variability of tacrolimus exposure in Chinese kidney transplant recipients. Transplant Proc. (2019) 51:1754–7. doi: 10.1016/j.transproceed.2019.04.019. PMID: 31255354

[12] Choi JS Ko H Kim HK Chung C Han A Min SK . Effects of tacrolimus intrapatient variability and CYP3A5 polymorphism on the outcomes of pediatric kidney transplantation. Pediatr Transplant. (2022) 26:e14297. doi: 10.1111/petr.14297. PMID: 35466485

[13] Chai Y Hu L Zhu Y Quan D Li Y Wang X . Association of CYP3A5 rs15524 and CYP3A7 rs776744 polymorphisms on tac IPV and clinical outcomes in early post-heart transplantation. BMC Med Genomics. (2025) 18:151. doi: 10.1186/s12920-025-02231-3. PMID: 41057866 PMC12505756

[14] Borra LC Roodnat JI Kal JA Mathot RA Weimar W van Gelder T . High within-patient variability in the clearance of tacrolimus is a risk factor for poor long-term outcome after kidney transplantation. Nephrol Dial Transplant. (2010) 25:2757–63. doi: 10.1093/ndt/gfq096. PMID: 20190242

[15] Sapir-Pichhadze R Wang Y Famure O Li Y Kim SJ . Time-dependent variability in tacrolimus trough blood levels is a risk factor for late kidney transplant failure. Kidney Int. (2014) 85:1404–11. doi: 10.1038/ki.2013.465. PMID: 24336032

[16] Soares ME Costa G Guerra L Morais MC Vaz N Codes L . Influence of tacrolimus intrapatient variability on allograft rejection frequency and survival following liver transplantation. Ther Drug Monit. (2024) 46:456–9. doi: 10.1097/ftd.0000000000001192. PMID: 38648652

[17] Wang X Liu Z Chen J Chai Y Shao X Xie W . Impact of intra-patient variability of tacrolimus on allograft function and CD4 + /CD8 + ratio in kidney transplant recipients: a retrospective single-center study. Int J Clin Pharm. (2024) 46:918–25. doi: 10.1007/s11096-024-01726-w. PMID: 38814512

[18] Kuypers DRJ . Intrapatient variability of tacrolimus exposure in solid organ transplantation: a novel marker for clinical outcome. Clin Pharmacol Ther. (2020) 107:347–58. doi: 10.1002/cpt.1618. PMID: 31449663

[19] Xie W Fan S Liu R Yan W Su C Zheng K . Tacrolimus intra-patient variability measures and its associations with allograft clinical outcomes in kidney transplantation. Transplant Rev (Orlando). (2024) 38:100842. doi: 10.1016/j.trre.2024.100842. PMID: 38537484

[20] Solomon S Colovai A Del Rio M Hayde N . Tacrolimus variability is associated with de novo donor-specific antibody development in pediatric renal transplant recipients. Pediatr Nephrol. (2020) 35:261–70. doi: 10.1007/s00467-019-04377-6. PMID: 31732803

[21] Lee H Min JW Kang H Lee H Eum SH Park Y . Combined analysis of HLA class II eplet mismatch and tacrolimus levels for the prediction of de novo donor specific antibody development in kidney transplant recipients. Int J Mol Sci. (2022) 23 (13):7357. doi: 10.3390/ijms23137357. PMID: 35806362 PMC9267119

[22] Del Bello A Gaible C Longlune N Hebral AL Esposito L Gandia P . Tacrolimus intrapatient variability after switching from immediate or prolonged-release to extended-release formulation, after an organ transplantation. Front Pharmacol. (2021) 12:602764. doi: 10.3389/fphar.2021.602764. PMID: 34690747 PMC8529208

[23] Mendoza Rojas A Hesselink DA van Besouw NM Dieterich M de Kuiper R Baan CC . High tacrolimus intrapatient variability and subtherapeutic immunosuppression are associated with adverse kidney transplant outcomes. Ther Drug Monit. (2022) 44:369–76. doi: 10.1097/ftd.0000000000000955. PMID: 35394988 PMC9083489

[24] Shuker N van Gelder T Hesselink DA . Intra-patient variability in tacrolimus exposure: causes, consequences for clinical management. Transplant Rev (Orlando). (2015) 29:78–84. doi: 10.1016/j.trre.2015.01.002. PMID: 25687818

[25] Susal C Dohler B . Late intra-patient tacrolimus trough level variability as a major problem in kidney transplantation: a collaborative transplant study report. Am J Transplant. (2019) 19:2805–13. doi: 10.1111/ajt.15346 30859672

[26] Nogueiras-Alvarez R Garcia-Saiz MDM . Tacrolimus intrapatient variability as a biomarker in solid organ transplantation. Clin Transplant. (2025) 39:e70197. doi: 10.1111/ctr.70197. PMID: 40504104

[27] Shuker N Shuker L van Rosmalen J Roodnat JI Borra LC Weimar W . A high intrapatient variability in tacrolimus exposure is associated with poor long-term outcome of kidney transplantation. Transpl Int. (2016) 29:1158–67. doi: 10.1111/tri.12798. PMID: 27188932

[28] Nuchjumroon A Vadcharavivad S Singhan W Poosoonthornsri M Chancharoenthana W Udomkarnjananun S . Comparison of tacrolimus intra-patient variability during 6–12 months after kidney transplantation between CYP3A5 expressers and nonexpressers. J Clin Med. (2022) 11(21):6320. doi: 10.3390/jcm11216320. PMID: 36362548 PMC9658797

[29] Phupradit A Vadcharavivad S Ingsathit A Kantachuvesiri S Areepium N Sra-Ium S . Impact of POR and CYP3A5 polymorphisms on trough concentration to dose ratio of tacrolimus in the early post-operative period following kidney transplantation. Ther Drug Monit. (2018) 40:549–57. doi: 10.1097/ftd.0000000000000542. PMID: 29878980

[30] Gu X-Q Tang D Wan P Qin T Yang T-H Wu J . Multiple microRNAs regulate tacrolimus metabolism through CYP3A5. Pharmacol Res. (2021) 164:105382. doi: 10.1016/j.phrs.2020.105382. PMID: 33348024

[31] Wan P Hou Y Qiu B Feng M Yang T Luo Y . GRWR correlates with the metabolism of tacrolimus after pediatric living donor liver transplantation according to donor CYP3A5 polymorphism. BioMed Res Int. (2022) 2022:7647754. doi: 10.1155/2022/7647754. PMID: 36349313 PMC9637468

[32] Viglietti D Loupy A Vernerey D Bentlejewski C Gosset C Aubert O . Value of donor-specific anti-HLA antibody monitoring and characterization for risk stratification of kidney allograft loss. J Am Soc Nephrol. (2017) 28:702–15. doi: 10.1681/asn.2016030368. PMID: 27493255 PMC5280026

[33] Gonzales HM McGillicuddy JW Rohan V Chandler JL Nadig SN Dubay DA . A comprehensive review of the impact of tacrolimus intrapatient variability on clinical outcomes in kidney transplantation. Am J Transplant. (2020) 20:1969–83. doi: 10.1111/ajt.16002. PMID: 32406604 PMC11140479

[34] Coste G Lemaitre F . The role of intra-patient variability of tacrolimus drug concentrations in solid organ transplantation: a focus on liver, heart, lung and pancreas. Pharmaceutics. (2022) 14(2):379. doi: 10.3390/pharmaceutics14020379. PMID: 35214111 PMC8878862

[35] You J Chen R Chai Y Wang X Xie W Yang Y . Comparing tacrolimus level monitoring in peripheral blood mononuclear cells and whole blood within one year after kidney transplantation: a single-center, prospective, observational study. Front Pharmacol. (2025) 16:1622702. doi: 10.3389/fphar.2025.1622702. PMID: 40567370 PMC12187596

[36] Sablik KA Clahsen-van Groningen MC Hesselink DA van Gelder T Betjes MGH . Tacrolimus intra-patient variability is not associated with chronic active antibody mediated rejection. PloS One. (2018) 13:e0196552. doi: 10.1371/journal.pone.0196552. PMID: 29746495 PMC5944964

[37] Kim EJ Kim SJ Huh KH Kim BS Kim MS Kim SI . Clinical significance of tacrolimus intra-patient variability on kidney transplant outcomes according to pre-transplant immunological risk. Sci Rep. (2021) 11:12114. doi: 10.1038/s41598-021-91630-4. PMID: 34108576 PMC8190283

[38] Kaya Aksoy G Comak E Koyun M Akbas H Akkaya B Aydinli B . Tacrolimus variability: a cause of donor-specific anti-HLA antibody formation in children. Eur J Drug Metab Pharmacokinet. (2019) 44:539–48. doi: 10.1007/s13318-019-00544-0. PMID: 30737655

[39] Lopez Del Moral C Wu K Naik M Osmanodja B Akifova A Lachmann N . The natural history of de novo donor-specific HLA antibodies after kidney transplantation. Front Med (Lausanne). (2022) 9:943502. doi: 10.3389/fmed.2022.943502 36186822 PMC9523126

[40] Loucks-DeVos JM Eagar TN Gaber AO Patel SJ Teeter LD Graviss EA . The detrimental impact of persistent vs an isolated occurrence of de novo donor-specific antibodies on intermediate-term renal transplant outcomes. Clin Transplant. (2017) 31 (8). doi: 10.1111/ctr.13025. PMID: 28582797

[41] Liu W Zhao J Kang ZY Xiao YL Yang L Liu C . De novo donor-specific HLA antibodies reduce graft survival rates and increase the risk of kidney transplant rejection: a single-center retrospective study. Transpl Immunol. (2021) 68:101430. doi: 10.1016/j.trim.2021.101430 34147608

[42] Betjes MGH Kal-van Gestel J Roodnat JI de Weerd AE . The incidence of antibody-mediated rejection is age-related, plateaus late after kidney transplantation, and contributes little to graft loss in the older recipients. Transpl Int. (2023) 36:11751. doi: 10.3389/ti.2023.11751. PMID: 38188697 PMC10768842

