# Supplementary material for: Timing matters: tacrolimus intra-patient variability within the initial seven months forecasts de novo DSA and subsequent rejection in a Chinese kidney transplant cohort
Source: Front Immunol. 2026 May 11;17:1809202. doi: 10.3389/fimmu.2026.1809202 (PMC13199030; doi:10.3389/fimmu.2026.1809202)
Supplement: Supplementary file 1 [file Table1.docx]

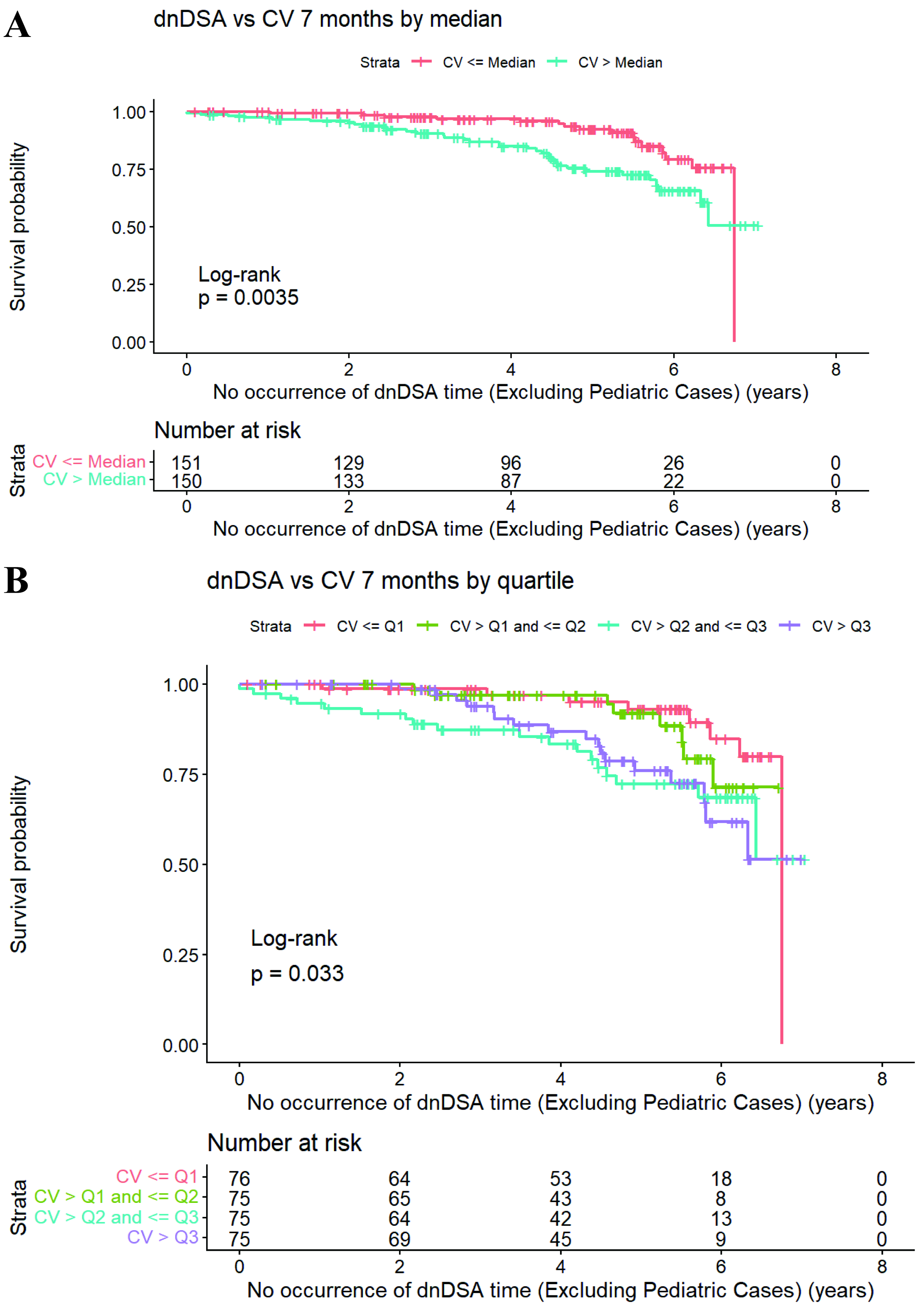


**Figure S1** Sensitivity analysis excluding pediatric recipients: effect of tacrolimus IPV on the occurrence of dnDSA in kidney transplant recipients at 7 months (n=409)

(A. stratified by the median CV%, B. stratified by the quartile CV% ; dnDSA:de novo donor-specific antibodies; CV: coefficient of variation)
